# Association between the CHG index and cardiometabolic multimorbidity: a nationwide prospective cohort and multi-community cross-sectional study

**DOI:** 10.3389/fnut.2025.1725495

**Published:** 2026-01-27

**Authors:** Li Liu, Yanyan Meng, Lianyi Hu, Runa A, Ying Zhang, Zhuye Gao

**Affiliations:** 1 Department of Cardiology, Xiyuan Hospital, China Academy of Chinese Medical Sciences, Beijing, China; 2 Beijing University of Traditional Chinese Medicine, Beijing, China; 3 Institute of Geriatric Medicine, Xiyuan Hospital, China Academy of Chinese Medical Sciences, Beijing, China; 4 Department of Internal Medicine, Yanke Hospital, China Academy of Chinese Medical Sciences, Beijing, China

**Keywords:** cardiometabolic diseases, cardiometabolic multimorbidity, cholesterol, high-density lipoprotein, glucose index, risk assessment

## Abstract

**Background:**

Cardiometabolic multimorbidity (CMM) is a growing public health challenge, with limited practical biomarkers available for early detection. Given the established relationship between impaired lipid as well as glucose homeostasis and CMM, this study aims to examine the association between the cholesterol, high-density lipoprotein, and glucose (CHG) index and CMM risk.

**Methods:**

A nationwide prospective cohort (CHARLS, 2011–2018; *n* = 8,425) and a multi-community cross-sectional cohort (Beijing, 2021; *n* = 1,734) were analyzed. In the CHARLS cohort, Cox proportional hazards models were used to assess the association between the CHG index and incident CMM. In both cohorts, logistic regression and restricted cubic splines (RCS) were employed to examine the cross-sectional association between CHG and CMM. Time-dependent receiver operating characteristic (ROC) curves evaluated predictive performance in CHARLS cohort, while conventional ROC analysis was used in the multi-community cohort. Subgroup analyses, interaction tests, and sensitivity analyses further evaluated result reliability.

**Results:**

In the CHARLS cohort, 491 (5.8%) individuals developed incident CMM over 7 years of follow-up. CHG levels were significantly elevated in patients with CMM (*p* < 0.001). Overall, the CHG index was associated with incident CMM (OR = 1.97, 95% CI: 1.53–2.53; HR = 1.82, 95% CI: 1.45–2.27). A nonlinear positive association was found in both cohorts. The CHG index demonstrated stable predictive accuracy over time (AUCs: 0.67 at 3 years, 0.64 at 5 years, 0.63 at 7 years). These findings were robustly validated in the multi-community cohort, where the CHG index was also significantly associated with prevalent CMM (OR = 2.82, 95% CI: 2.04–3.90). Subgroup analyses confirmed robustness across populations, especially among those without baseline cardiometabolic disease.

**Conclusion:**

An elevated CHG index is independently associated with an increased risk of CMM across both prospective and cross-sectional studies. It serves as a robust and reproducible predictor for the early identification of CMM in diverse Chinese populations.

## Graphical abstract

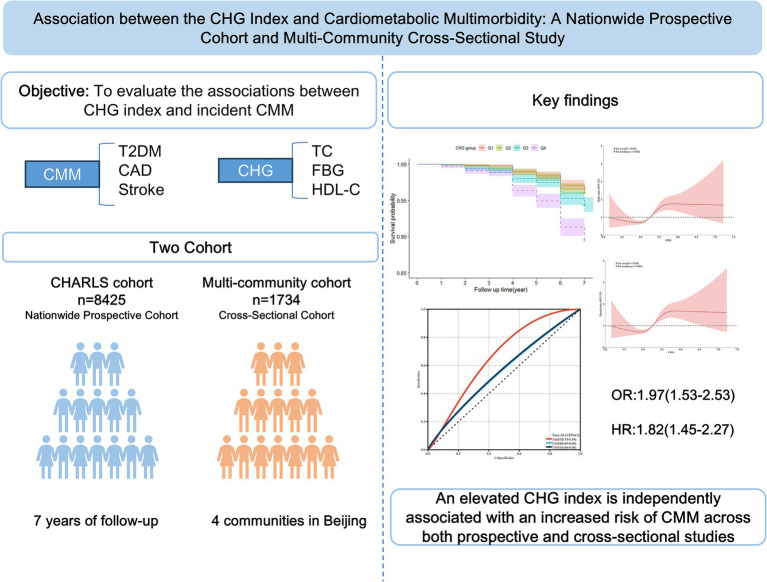

## 1 Introduction

Cardiometabolic diseases (CMDs) affect approximately half of American adults and represent major risk factors for hospitalization and mortality (1). Cardiometabolic multimorbidity (CMM) refers to the co-existence of at least two CMD, including type two diabetes (T2DM), coronary artery disease (CAD), and stroke (2). As one of the most common and severe multimorbidities, CMM can adversely affect health status in older individuals, undermining the quality of life and impairing self-rated health (2, 3). Furthermore, CMM has been linked to adverse health outcomes, such as cognitive impairment (4), dementia (5), and functional disabilities (6). Critically, it confers a significant increase in all-cause mortality risk compared to single CMD (7). Nevertheless, clinically accessible and cost-effective biomarkers for the diagnosis of early-stage CMM remain lacking.

Recently, research interest in risk factors of CMM has grown rapidly worldwide. Given their role in the development of different cardiometabolic diseases and their progression to multimorbidity, obesity, dyslipidemia, and insulin resistance (IR) have been suggested as key risk factors for multimorbidity (8–10). Emerging diagnostic markers primarily target these mechanisms and possess preliminary diagnostic efficacy (9, 11–13); however, due to differences in study designs and patient characteristics, there is no consensus regarding optimal indices for predicting CMM. The cholesterol, high-density lipoprotein, and glucose (CHG) index, derived from total cholesterol (TC), fasting blood glucose (FBG), and high-density lipoprotein cholesterol (HDL-C) (14), is proposed to represent an integrated measure of impaired lipid and glucose homeostasis. This index is useful for detecting type 2 diabetes mellitus (T2DM) and shows superior diagnostic application for T2DM compared to the triglyceride-glucose (TyG) index (14). Moreover, the CHG index also positively correlates with cardiovascular disease risk, exhibiting predictive capacity comparable to the TyG (15). Although the CHG index has shown considerable diagnostic efficacy and predictive ability in CMDs, it remains uncertain whether the CHG index can reliably predict CMM.

There are no large population-based studies exploring the longitudinal relationship between the CHG index and CMM among middle-aged and older adults. Using a large-scale, nationally representative prospective cohort (CHARLS, 2011–2018) and a multi-community cross-sectional cohort (Beijing, 2021), we conducted a study with 7 years of follow-up to investigate the association between the CHG index and CMM, aiming to evaluate the validity of the CHG index as a predictor of CMM and provide evidence for early prevention and diagnosis.

## 2 Materials and methods

### 2.1 Study population

The CHARLS, established in 2011, is an ongoing nationally representative longitudinal survey in China. It collects high-quality data through one-to-one interviews with a structured questionnaire, from a nationally representative sample of Chinese population aged ≥45 years old, selected via multistage stratified probability-proportionate-to-size sampling. All participants underwent an assessment using a standardized questionnaire to collect data on sociodemographic and lifestyle factors and health-related information. In wave 1(2011–2012), a total of 17,708 participants across 150 counties/districts and 450 villages within 28 provinces were recruited and followed through wave 2 (2013–2014), Wave 3 (2015–2016), and Wave 4 (2017–2018). The data included individual weighting variables to ensure that the survey sample was nationally representative. Detailed CHARLS study design information has been previously re-ported (16). All participants provided informed consent, and the protocol was approved by the Ethical Review Committee of Peking University (approval number: IRB00001052–11,015). All procedures complied with institutional/national ethical standards, the Declaration of Helsinki (1964, amended), and comparable standards. This study fol-lowed the STROBE reporting guideline (17).

The multi-community cohort consists of residents from four communities in Haidian District, Beijing, who participated in screening between September 2020 and November 2022. A total of 2,337 individuals completed data collection for this study, which was approved by the Ethics Committee of the China Academy of Chinese Medical Sciences (No. 2021XLA001-3), and all participants signed informed consent.

**Figure 1:**
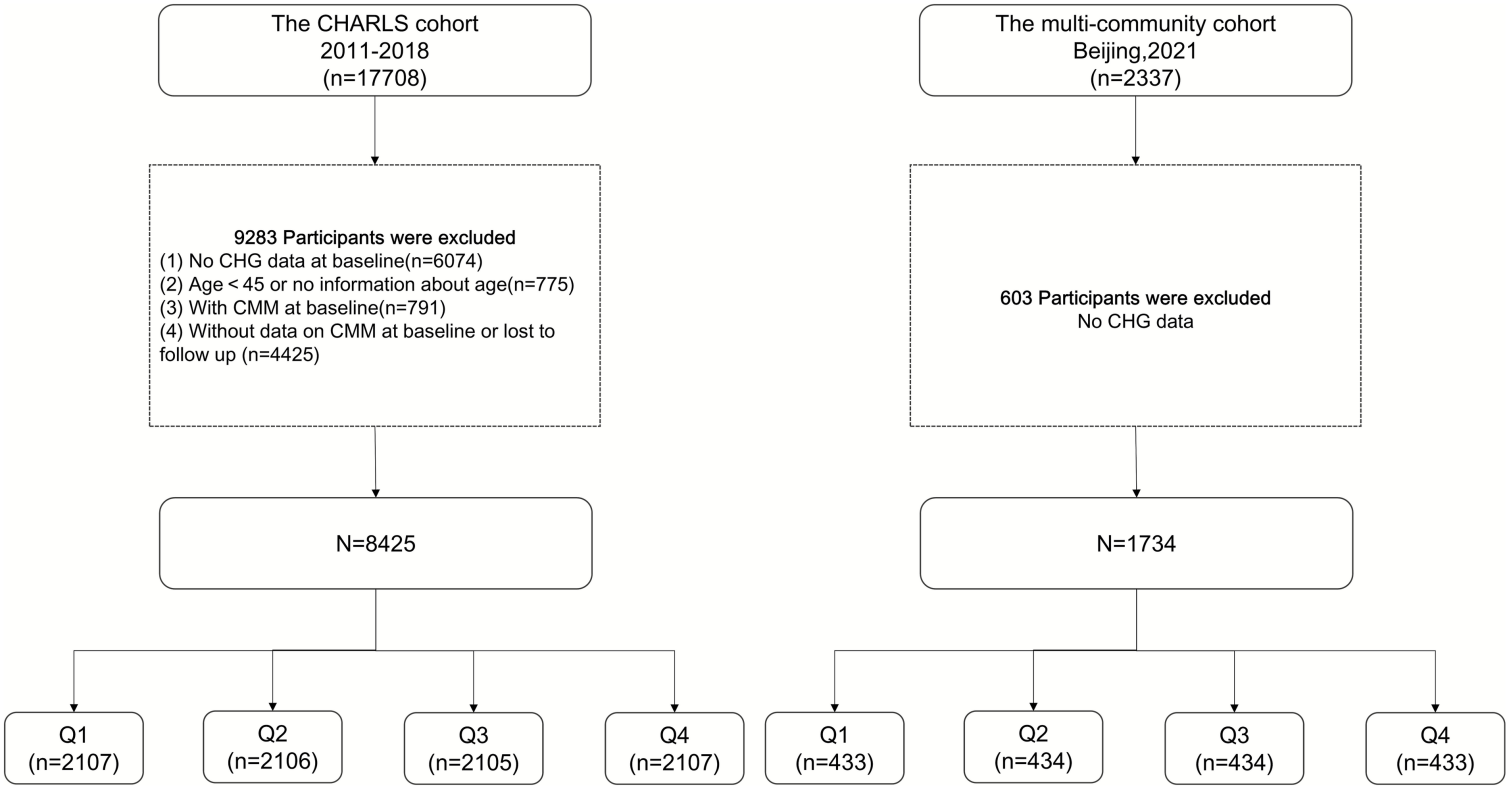
Flow chart of sample selection and the exclusion criteria. CHG, cholesterol, high-density lipoprotein, and glucose index; CMM, cardiometabolic multimorbidity.

In the CHARLS cohort, we retrospectively analyzed data from 17,708 participants in wave 1. We excluded 9,283 participants due to the following reasons: no CHG data at baseline (*n* = 6,074), age <45 years or missing age data (*n* = 775), baseline CMM (*n* = 791), and missing baseline CMM data or loss to follow-up (*n* = 4,425). In the multi-community cohort, 603 participants were excluded due to the absence of CHG data. As a result, 8,425 participants from the CHARLS cohort and 1,734 participants from the multi-community cohort were included in the analysis. Figure 1 illustrates the detailed selection process.

### 2.2 CHG index assessment

The blood testing was conducted at the Youanmen Center for Clinical Laboratory at Capital Medical University. The samples analyzed were derived from frozen plasma or whole blood, utilizing an enzymatic colorimetric assay method. Measurement details for TC, FBG, HDL-C, and other indicators are provided in Supplementary material 1. The formula for calculating the CHG index is: CHG index = Ln [TC (mg/dL) × FBG (mg/dL)/2 × HDL-C (mg/dL)] (14).

### 2.3 Assessment of CMM

CMM events were defined as the simultaneous presence of at least two cardiometabolic diseases (CMDs), including diabetes, heart disease and stroke. Ascertainment of physician-diagnosed chronic conditions was based on self-reports at baseline and in follow-up surveys (for diabetes or high blood sugar, “Have you been diagnosed with diabetes or high blood sugar by a doctor?,” for heart attack, coronary heart disease, or other heart problems, “Have you been diagnosed with heart attack, coronary heart disease, angina, congestive heart failure, or other heart problems by a doctor?” and for stroke, “Have you been diagnosed with stroke by a doctor?”). If the participant’s answer to the question was “Yes,” they were considered to have a cardiometabolic condition. Of those, CMM was defined as participants who reported suffering from at least two cardiometabolic conditions. The incidence of CMM was determined at the time of diagnosis of the second CMM diagnosis, at which point the individuals presented with two distinct types of cardiometabolic diseases.

In addition to self-reporting diabetes, participants were diagnosed with diabetes if they met any of the following criteria: to define diabetes, criteria included an FBG level reaching 126 mg/dL or more, a 2-h post-challenge glucose level of ≥11.1 mmol/L, HbA1c at 48 mmol/mol (6.5%) or higher, or the administration of antidiabetic drugs, according to the American Diabetes Association criteria (18).

### 2.4 Assessments of covariates

Trained interviewers collected information on socio-demographic status and health-related factors using a structured questionnaire. Sociodemographic variables included age, sex, residence (rural, urban), marital status (married and others), and education level (primary school and below, secondary school and higher). Health-related factors included body mass index (BMI), smoking and drinking status (yes or no), diabetes, heart disease, stroke, hypertension, dyslipidemia, and use of medications for diabetes, heart disease, stroke.

Considering that baseline cardiometabolic diseases could also affect the results, we set the variable CMD 2011 to represent the conditions of participants at the time of enrollment. When CMD 2011 = 0, the participant was free of cardiometabolic diseases at baseline, and when CMD 2011 = 1, the participant was diagnosed with one cardiometabolic disease at baseline.

### 2.5 Statistical analysis

Data are presented as means ± standard deviation (SD) or median and interquartile range for continuous variables and percentages for categorical variables. Baseline characteristics were summarized based on CMM status and CHG index levels, com-pared between groups using the chi-squared test, analysis of variance, Tukey’s test or the Kruskal-Wallis test, as appropriate. The quartile ranges for CHG index were as fol-lows: Q1:≤5.02, Q2:5.03–5.26, Q3: 5.27–5.52, and Q4 ≥ 5.53 (CHARLS cohort); Q1: ≤ 4.84, Q2:4.85–5.06, Q3: 5.07–5.31, and Q4 ≥ 5.32 (Multi-community cohort).

Kaplan–Meier survival curves were employed to facilitate a more intuitive analysis of the cumulative risk of CMM associated with the CHG index over time. In constructing the model, we defined the time axis at 1–7 years observation period, and the vertical axis represented the survival probability. To further examine the relationship between the CHG index and CMM risk, we established both univariate and multivariate logistic and Cox regressions, with adjustments for confounding factors likely to impact the results. The selection of covariates was based on previous studies and statistical methods, with careful consideration of factors that may influence the outcome. Three models were estimated: in model 1 unadjusted for any covariates; in model 2 adjusted for gender, age, residence, marital status, education level, smoking status, and drinking status; in model 3 adjusted for gender, age, residence, marital status, education level, smoking status, drinking status, diabetes, heart disease, stroke, hypertension, dyslipidemia, diabetes medications, heart disease medications, stroke medications, status of CMD 2011, SBP, DBP, and BMI. Prior to conducting the Cox regression model, proportional hazards assumption was verified using Schoenfeld residuals.

Restricted cubic spline (RCS) function analysis was performed to fit the model and visualize the relationship between CHG index and the risk of developing CMM. The adjustment factors for these three RCS models were the same with above. Additionally, we performed ROC analysis using the R software package pROC (version 1.17.0.1) to obtain the AUC. Specifically, we obtained the follow-up time of participants and CHG index, and used the roc function of pROC to perform ROC analyses at time points 3, 5, and 7 years. The ci function of pROC was used to evaluate AUC and confidence intervals to obtain the final AUC results (19). Subgroup analyses were performed to explore whether the association between CHG index with CMM differed by gender, age, residential, marital status, education level, smoking status, drinking status, status of CMM. All statistical analysis was performed retrospectively with R version 4.5.0 (R Foundation for Statistical Computing). All comparisons were two-sided, and a signific.

## 3 Results

### 3.1 Participants characteristics

In total, 8,425 participants of CHARLS and 1734 participants of multi-community cohort were included for analysis based on the inclusion and exclusion criteria. Figure 1 depicts the overall methodological workflow of the present study. Over 7 years of follow-up, 491 (5.8%) participants were identified with new-onset CMM. In CHARLS cohort, compared to the non-CMM group, the CMM group comprised older participants, had a higher proportion of females and urban residents, exhibited elevated levels of BMI, SBP, DBP, hsCRP, FBG, TC, TG, LDL-C, HbA1c, UA, and CHG (*p* < 0.05). At baseline, participants in the CMM group were more likely to have diabetes, heart disease, stroke, hypertension, and dyslipidemia and consume medications for diabetes, heart disease, and stroke. Conversely, individuals in the CMM group had a lower prevalence of smoking and lower levels of HDL-C (*p* < 0.05) (Table 1). In the multi-community cohort, the CMM prevalence was 18.17%. There were no differences in gender, but differences were observed in marital status and education level. DBP and TG levels did not differ, while other trends were consistent with the CHARLS cohort (Supplementary Table S1).

**Table 1:** Baseline characteristics of the study participants according to cardiometabolic multimorbidity outcomes (CHARLS cohort).

Characteristic	Overall	Non-CMM group	CMM group	*p*-value
*N*	8,425	7,934	491	
Age, year	58.36 ± 8.86	58.24 ± 8.87	60.43 ± 8.34	<0.001
Man, n(%)	3,863(45.9)	3,669(46.2)	194(39.5)	0.004
Rural (vs. urban), n(%)	5,626(66.8)	5,325(67.1)	301(61.3)	0.008
Current married, n(%)	7,109(84.4)	6,695(84.4)	414(84.3)	0.969
Educational level, n(%)				0.784
Primary school or lower	7,614(90.4)	7,172(90.4)	442(90.0)	
Secondary school or higher	811(9.6)	762(9.6)	49(10.0)	
Smoking, n(%)	3,222(38.2)	3,057(38.5)	165(33.6)	0.029
Drinking, n(%)	3,268(38.8)	3,084(38.9)	184(37.5)	0.538
Basal diabetes, n(%)	143(1.7)	112(1.4)	31(6.3)	<0.001
Basal heart disease, n(%)	791(9.4)	625(7.9)	166(33.8)	<0.001
Basal stroke, n(%)	118(1.4)	94(1.2)	24(4.9)	<0.001
Basal hypertension, n(%)	2036(24.2)	1758(22.2)	278(56.6)	<0.001
Basal dyslipidemia, n(%)	687(8.2)	586(7.4)	101(20.6)	<0.001
Diabetes medications, n(%)	79(0.9)	64(0.8)	15(3.1)	<0.001
Heart disease medications, n(%)	542(6.4)	440(5.5)	102(20.8)	<0.001
Stroke medications, n(%)	67(0.8)	50(0.6)	17(3.5)	<0.001
BMI, kg/m^2^	24.08 ± 4.93	23.94 ± 4.86	26.30 ± 5.57	<0.001
SBP, mmHg	128.30 ± 20.93	127.74 ± 20.69	137.25 ± 22.52	<0.001
DBP, mmHg	75.01 ± 12.05	74.77 ± 11.98	78.92 ± 12.49	<0.001
hsCRP, mg/dL	0.92(0.51, 1.82)	0.90(0.51, 1.78)	1.31(0.69, 2.50)	<0.001
FBG, mg/dL	101.52(93.96, 110.34)	101.16(93.78, 109.80)	106.02(97.92, 117.32)	<0.001
TC, mg/dL	189.82(166.62, 214.56)	189.05(166.62, 213.79)	196.97(170.97, 224.32)	0.001
TG, mg/dL	103.55(74.34, 149.57)	101.78(73.45, 147.50)	124.79(91.16, 177.00)	<0.001
LDL-C, mg/dL	114.05(93.56, 136.86)	113.66(93.17, 136.08)	121.01(94.43, 146.42)	<0.001
HDL-C, mg/dL	49.87(40.59, 59.92)	49.87(40.98, 60.31)	46.01(37.50, 54.51)	<0.001
HbA1c, %	5.10(4.90, 5.40)	5.10(4.90, 5.40)	5.20(5.00, 5.50)	<0.001
UA, mg/dL	4.28(3.56, 5.11)	4.26(3.55, 5.09)	4.39(3.68, 5.25)	0.004
BUN, mg/dL	15.13(12.55, 18.18)	15.15(12.55, 18.21)	14.79(12.35, 17.67)	0.068
CHG	5.26(5.02, 5.52)	5.25(5.01, 5.50)	5.46(5.18, 5.71)	<0.001

Date are presented as median (25th to 75th interquartile range) or n (%). CHG, cholesterol, high-density lipoprotein, and glucose index; CMM, cardiometabolic multimorbidity; BMI, body mass index; SBP, systolic blood pressure; DBP, diastolic blood pressure; hsCRP, high-sensitivity C-reactive protein; FBG, fasting plasma glucose; TC, total cholesterol; TG, triglycerides; LDL-C, low-density lipoprotein cholesterol; HDL-C, high-density lipoprotein cholesterol; HbA1c, glycosylated hemoglobin A1c; UA, uric acid; BUN, blood urea nitrogen.

### 3.2 Baseline characteristics based on the quantiles of CHG

In CHARLS cohort, the participants were divided into four groups based on the quartiles of the CHG index (Table 2). The incidence of CMM in quartiles Q1, Q2, Q3, and Q4 were 3.4, 3.8, 5.7, and 10.4%, respectively (*p* < 0.001). Participants in the higher quantile of CHG were more likely to be urban residents and married, and exhibited higher levels of BMI, SBP, DBP, hsCRP, FBG, TC, TG, LDL-C, HbA1c, UA, and BUN (*p* < 0.05). Additionally, individuals in the higher quantile of the CHG index were more likely to have diabetes, hypertension, dyslipidemia, and CMM at baseline (*p* < 0.05). In contrast, the prevalence of drinking and HDL-C levels was lower in the higher quantile of the CHG index (*p* < 0.05). In multi-community cohort, the incidence of CMM in quartiles Q1, Q2, Q3, and Q4 were 12.9, 16.1, 15.9, and 27.7%, respectively (*p* < 0.001) (Supplementary Table S2). Except for marital status, drinking, and dyslipidemia, the trends in other variables were consistent with those observed in the CHARLS cohort.

**Table 2:** Baseline characteristics of participants according to the quartiles of CHG (CHARLS cohort).

Characteristic	Total	CHG quartiles				*P*-value
		Q1	Q2	Q3	Q4	
*N*	8,425	2,107	2,106	2,105	2,107	
Age, year	58.36 ± 8.86	58.22 ± 9.14	58.34 ± 8.97	58.30 ± 8.78	58.60 ± 8.52	0.547
Man, n(%)	3,863(45.9)	1,014(48.1)	950(45.1)	940(44.7)	959(45.5)	0.104
Rural(vs urban), n(%)	5,626(66.8)	1,521(72.2)	1,472(69.9)	1,362(64.7)	1,271(60.3)	<0.001
Current married, n(%)	7,109(84.4)	1748(83.0)	1771(84.1)	1775(84.3)	1815(86.1)	0.040
Educational level, n(%)						0.456
Primary school or lower	7,614(90.4)	1920(91.1)	1907(90.6)	1896(90.1)	1891(89.7)	
Secondary school or higher	811(9.6)	187(8.9)	199(9.4)	209(9.9)	216(10.3)	
Smoking, n(%)	3,222(38.2)	855(40.6)	797(37.8)	779(37.0)	791(37.5)	0.078
Drinking, n(%)	3,268(38.8)	901(42.8)	816(38.7)	780(37.1)	771(36.6)	<0.001
Basal Diabetes, n(%)	143(1.7)	27(1.3)	27(1.3)	49(2.3)	40(1.9)	0.020
Basal heart disease, n(%)	791(9.4)	194(9.2)	185(8.8)	203(9.6)	209(9.9)	0.605
Basal stroke, n(%)	118(1.4)	26(1.2)	26(1.2)	30(1.4)	36(1.7)	0.512
Basal Hypertension, n(%)	2036(24.2)	368(17.5)	441(20.9)	528(25.1)	699(33.2)	<0.001
Basal Dyslipidemia, n(%)	687(8.2)	81(3.8)	148(7.0)	168(8.0)	290(13.8)	<0.001
Diabetes medications, n(%)	79(0.9)	18(0.9)	16(0.8)	19(0.9)	26(1.2)	0.408
Heart disease medications, n(%)	542(6.4)	143(6.8)	128(6.1)	133(6.3)	138(6.5)	0.808
Stroke medications, n(%)	67(0.8)	14(0.7)	17(0.8)	18(0.9)	18(0.9)	0.885
BMI, kg/m^2^	24.08 ± 4.93	22.59 ± 4.72	23.60 ± 4.78	24.58 ± 4.86	25.56 ± 4.88	<0.001
SBP, mmHg	128.30 ± 20.93	125.05 ± 21.03	126.80 ± 20.04	129.63 ± 21.34	131.74 ± 20.64	<0.001
DBP, mmHg	75.01 ± 12.05	73.11 ± 12.45	73.99 ± 11.60	75.98 ± 12.01	76.97 ± 11.85	<0.001
hsCRP, mg/dL	0.92(0.51, 1.82)	0.70(0.41, 1.47)	0.80(0.48, 1.57)	0.97(0.57, 1.84)	1.25(0.71, 2.42)	<0.001
FBG, mg/dL	101.52(93.96, 110.34)	93.96(86.94, 100.62)	99.36(93.06, 106.20)	102.96(96.66, 110.88)	111.42(102.96, 145.44)	<0.001
TC, mg/dL	189.82(166.62, 214.56)	171.65(152.70, 194.07)	185.95(165.08, 207.22)	193.69(172.04, 216.11)	208.76(184.79, 238.53)	<0.001
TG, mg/dL	103.55(74.34, 149.57)	71.69(56.64, 92.93)	90.27(70.80, 118.59)	115.94(88.50, 152.22)	172.58(123.90, 238.95)	<0.001
LDL-C, mg/dL	114.05(93.56, 136.86)	95.10(80.80, 111.34)	114.05(97.42, 131.06)	123.71(104.38, 143.82)	128.74(102.45, 156.19)	<0.001
HDL-C, mg/dL	49.87(40.59, 59.92)	63.02(54.90, 73.84)	53.74(47.17, 61.47)	46.39(40.59, 52.96)	37.50(32.47, 44.07)	<0.001
HbA1c, %	5.10(4.90, 5.40)	5.00(4.80, 5.30)	5.10(4.80, 5.30)	5.10(4.90, 5.40)	5.20(4.90, 5.50)	<0.001
UA, mg/dL	4.28(3 0.56, 5.11)	4.02(3.40, 4.85)	4.16(3.46, 4.91)	4.26(3.57, 5.11)	4.64(3.90, 5.53)	<0.001
BUN, mg/dL	15.13(12.55 18.18)	15.46(12.55, 18.68)	15.07(12.58, 12.21)	14.96(12.49, 17.98)	14.96(12.55, 17.87)	0.008
CMM, n(%)	491(5.8)	71(3.4)	81(3.8)	119(5.7)	220(10.4)	<0.001

Date are presented as median (25th to 75th interquartile range) or n (%). CHG, Cholesterol, High density lipoprotein, and Glucose index; CMM, cardiometabolic multimorbidity; BMI, body mass index; SBP, systolic blood pressure; DBP, diastolic blood pressure; hsCRP, high-sensitivity C-reactive protein; FBG, fasting plasma glucose; TC, total cholesterol; TG, triglycerides; LDL-C, low-density lipoprotein cholesterol; HDL-C, high-density lipoprotein cholesterol; HbA1c, glycosylated hemoglobin A1c; UA, uric acid; BUN, blood urea nitrogen.

### 3.3 Relationship between CHG and the incidence of CMM

**Figure 2:**
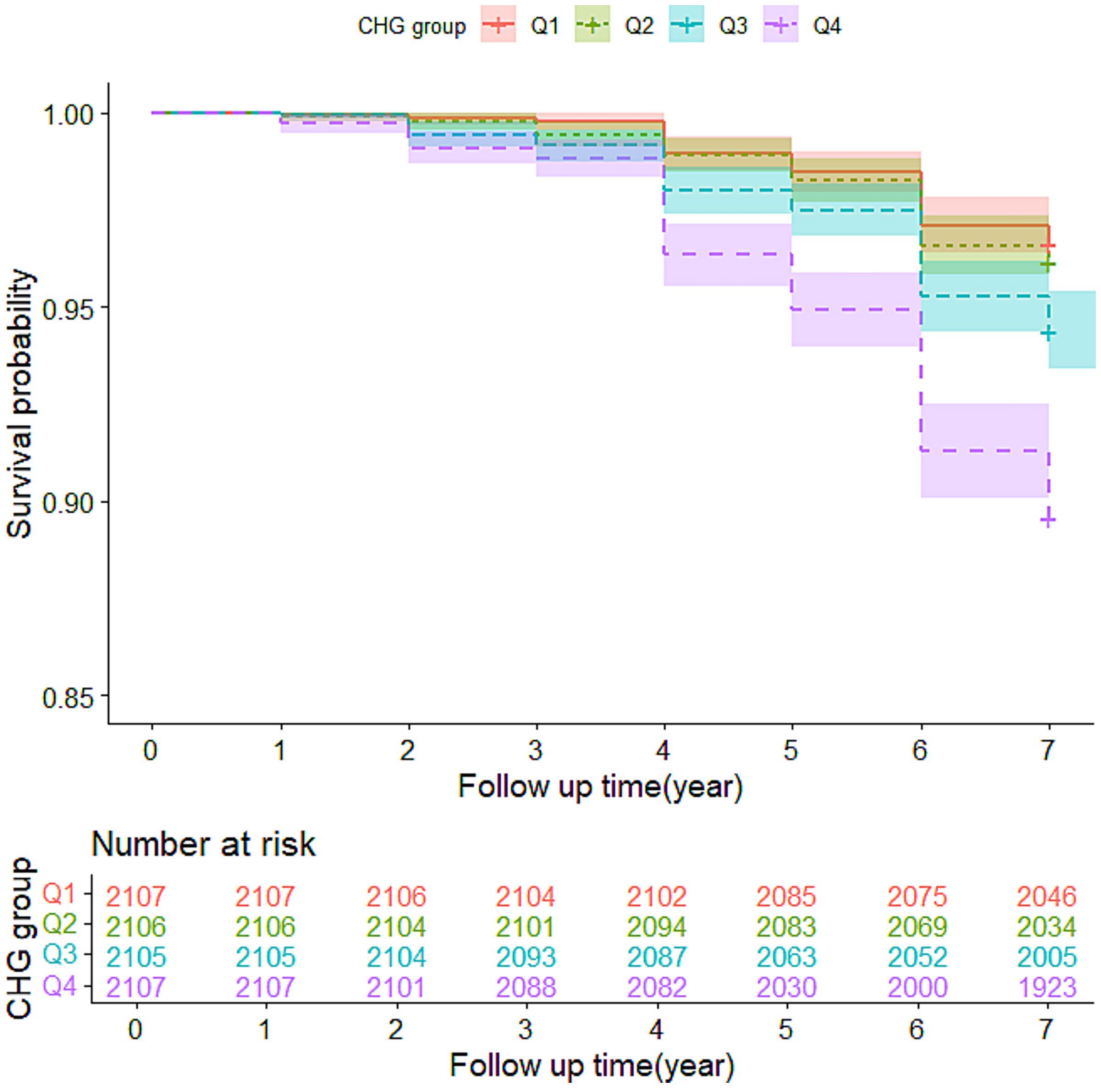
Kaplan–Meier curves for the survival probability of CMM (CHARLS cohort) Abbreviation: CHG, cholesterol, high-density lipoprotein, and glucose index; CMM, cardiometabolic multimorbidity.

The survival probability of CMM was analyzed using Kaplan–Meier survival curve. The survival probability of CMM decreased gradually with increasing CHG across the quartiles (log-rank = 7, *p* < 0.001) (Figure 2). Logistic regression analysis was conducted to validate the association between baseline CHG index and the risk of CMM both CHARLS cohort and multi-community cohort. In CHARLS cohort, when treating CHG as a continuous variable, a higher CHG was significantly associated with an increased risk of CMM, both before and after adjustment for other confounding covariates (OR = 2.63, 95% CI: 2.14–3.23, *p* < 0.001 in model 1; OR = 2.61, 95% CI: 2.11–3.21, *p* < 0.001 in model 2; and OR = 1.97, 95% CI: 1.53–2.53, *p* < 0.001 in model 3). In addition, individuals in quartiles Q3 and Q4 showed a significantly increased risk of CMM (Q3 vs. Q1: OR = 1.47, *P* < 0.05 in model 3; Q4 vs. Q1: OR = 2.40, *p* < 0.001 in model 3). In the multi-community cohort, the association between baseline CHG index and the risk of CMM was also significant (OR = 2.82, 95% CI: 2.04–3.90, *p* < 0.001 in model 3). The CHG level in Q4 was significantly higher than in Q1 (OR = 2.55, 95% CI: 1.78–3.67, *p* < 0.001 in model 3) (Table 3).

**Table 3:** Logistic regression analysis of baseline CHG and CMM (CHARLS cohort and Multi-community cohort).

Quartiles of CHG	OR (95% CI)		
	Model 1^a^	Model 2^b^	Model 3^c^
CHARLS cohort			
CHG, continuous	2.63(2.14–3.23)***	2.61(2.11–3.21)***	1.97(1.53–2.53)***
CHG, categories			
Q1	Reference	Reference	Reference
Q2	1.15(0.83–1.59)	1.13(0.82–1.57)	1.09(0.78–1.53)
Q3	1.72(1.28–2.33)***	1.69(1.25–2.29)***	1.47(1.07–2.02)*
Q4	3.34(2.55–4.43)***	3.25(2.48–4.31)***	2.40(1.80–3.26)***
Community cohort			
CHG, continuous	2.95(2.15–4.05)***	2.98(2.16–4.12)***	2.82(2.04–3.90)***
CHG, categories			
Q1	Reference	Reference	Reference
Q2	1.29 (0.89–1.89)	1.36 (0.93–2.01)	1.31(0.89–1.94)
Q3	1.27(0.87–1.86)	1.32 (0.89–1.94)	1.24(0.84–1.84)
Q4	2.58(1.82–3.67)***	2.72(1.90–3.89)***	2.55 (1.78–3.67)***

CHARLS cohort: ^a^Model 1 unadjusted for any covariates, ^b^Model 2 adjusted for gender, age, residence, marital status, education level, smoking status, and drinking status, ^c^Model 3 adjusted for gender, age, residence, marital status, education level, smoking status, drinking status, diabetes, heart disease, stroke, hypertension, dyslipidemia, diabetes medications, heart disease medications, stroke medications, status of CMD 2011, SBP, DBP, and BMI. Community cohort: ^a^Model 1 unadjusted for any covariates, ^b^Model 2 adjusted for gender, age, marital status, education level, smoking status, and drinking status, ^c^Model 3 adjusted for gender, age, residence, marital status, education level, smoking status, drinking status, SBP, DBP, and BMI. CHG, cholesterol, high-density lipoprotein, and glucose index. *p < 0.05. **p < 0.01. ***p < 0.001.

The proportional hazards assumption using the Schoenfeld residual test showed no violations (*p* > 0.05). Table 4 summarizes the association of the CHG index as a continuous variable and categorical variable with the risk of CMM (HR = 2.39, 95% CI = 2.01–2.85, *p* < 0.001 in model 1; HR = 2.39, 95% CI = 2.00–2.87, *p* < 0.001 in model 2; and HR = 1.82, 95% CI = 1.45–2.27, *p* < 0.001 in model 3). Individuals in quartiles Q3 and Q4 showed a significantly increased risk of CMM in multi-adjusted Cox regression analysis (Q3 vs. Q1: HR = 1.47, *P* < 0.05 in model 3; Q4 vs. Q1: HR = 2.40, *p* < 0.001 in model 3).

**Table 4:** Cox proportional hazards regression model analysis of baseline CHG and CMM (CHARLS cohort).

Quartiles of CHG	HR (95% CI)		
	Model 1^a^	Model 2^b^	Model 3^c^
CHG, continuous	2.39(2.01–2.85)***	2.39(2.00–2.87)***	1.82(1.45–2.27)***
CHG, categories			
Q1	Reference	Reference	Reference
Q2	1.15(0.83–1.57)	1.31(0.82–1.55)	1.09(0.80–1.51)
Q3	1.72(1.25–2.26)***	1.68(1.23–2.22)***	1.47(1.06–1.92)*
Q4	3.34(2.46–4.21)***	3.25(2.38–4.08)***	2.40(1.68–2.94)***

^a^Model 1 unadjusted for any covariates. ^b^Model 2 adjusted for gender, age, residence, marital status, education level, smoking status, and drinking status. ^c^Model 3 adjusted for gender, age, residence, marital status, education level, smoking status, drinking status, diabetes, heart disease, stroke, hypertension, dyslipidemia, diabetes medications, heart disease medications, stroke medications, status of CMD 2011, SBP, DBP, and BMI. CHG, cholesterol, high-density lipoprotein, and glucose index. *p < 0.05. **p < 0.01. ***p < 0.001.

**Figure 3:**
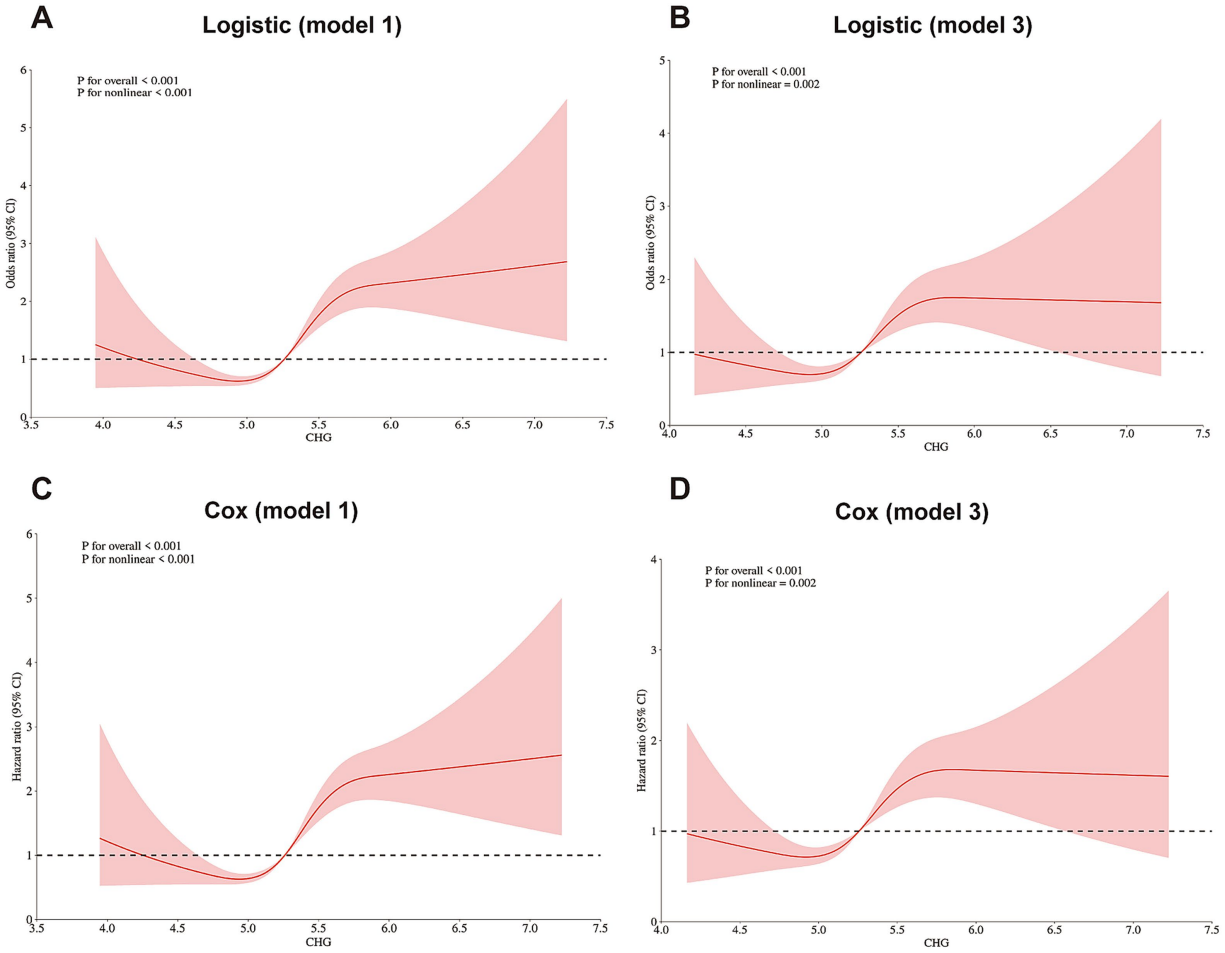
Restricted cubic spline curves for CMM according to CHG using logistic regression analysis **(A,B)** and Cox proportional hazards regression model analysis **(C,D)**, without **(A,C)** or with **(B,D)** adjustment for gender, age, residence, marital status, education level, smoking status, drinking status, diabetes, heart disease, stroke, hypertension, dyslipidemia, diabetes medications, heart disease medications, stroke medications, status of CMD 2011, SBP, DBP, and BMI *(CHARLS cohort)*. CHG, cholesterol, high-density lipoprotein, and glucose index; CMM, cardiometabolic multimorbidity.

Additionally, RCS were conducted to model and visualize the relationship between the CHG index and the risk of CMM. A positive nonlinear association was found between the CHG index and the risk of CMM (Figure 3; Supplementary Figure S1), even after adjusting for the confounding covariates (*P* for overall < 0.001, *P* for nonlinear < 0.001).

### 3.4 Discriminative power analysis

**Figure 4:**
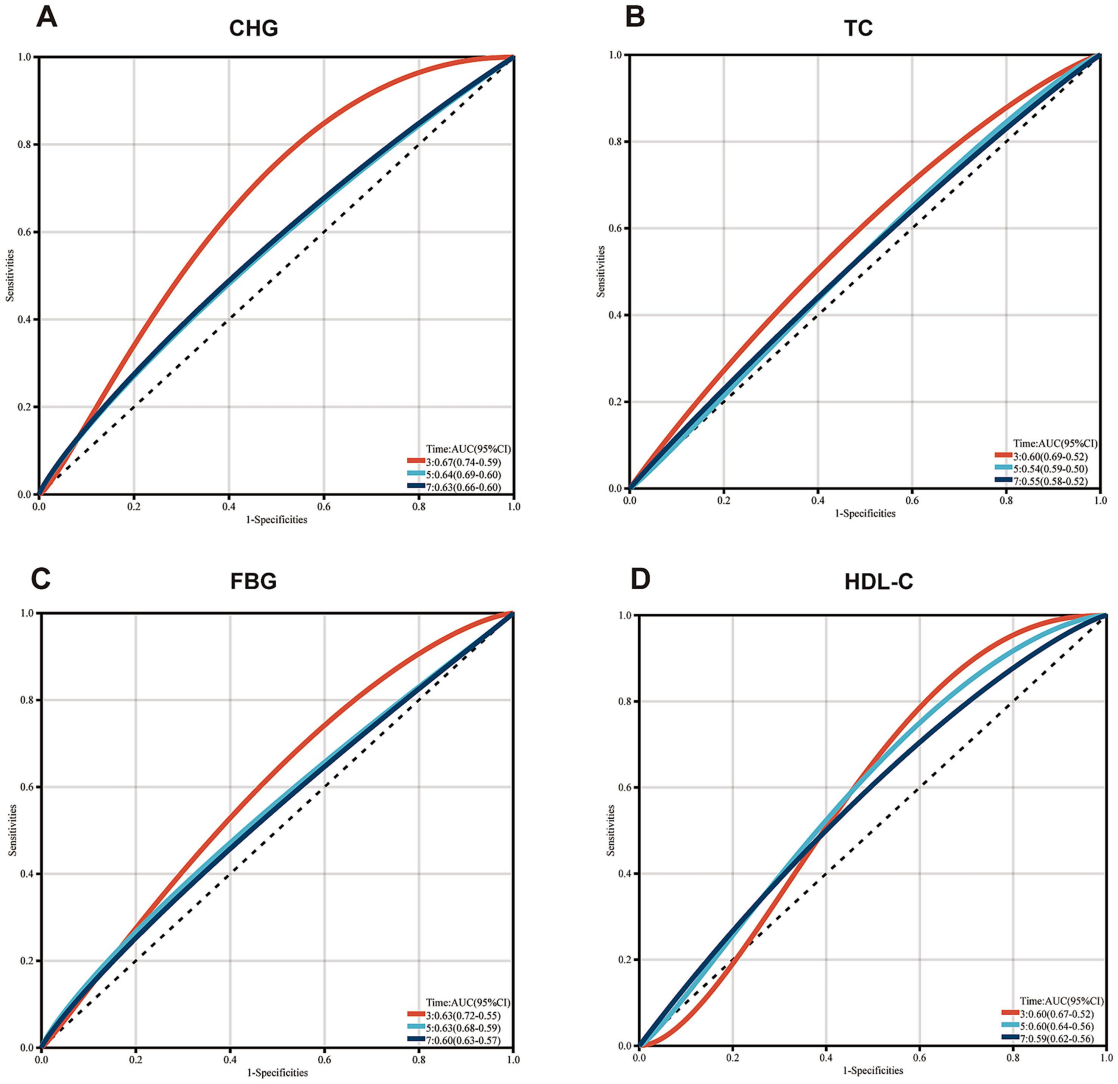
Receiver-operating characteristic curves for prediction of CMM *(CHARLS cohort)*. AUC, area under the curve; CHG, cholesterol, high-density lipoprotein, and glucose index; TC, total cholesterol; FBG, fasting plasma glucose; HDL-C, high-density lipoprotein cholesterol.

ROC analysis was conducted to assess and compare the predictive abilities of CHG, TC, FBG, and HDL-C. In CHARLS cohort, time-dependent ROC curves exhibited consistent predictive accuracy of CHG at 3 years (AUC 0.67, 95% CI: 0.59–0.74), 5 years (AUC 0.64, 95% CI: 0.60–0.69), and 7 years (AUC 0.63, 95% CI: 0.60–0.66) (Figure 4A). Time-dependent ROC curves exhibited the predictive accuracy of TC at 3 years (AUC 0.60, 95% CI = 0.52–0.69), 5 years (AUC 0.54, 95% CI = 0.52–0.58), and 7 years (AUC 0.55, 95% CI = 0.52–0.58) (Figure 4B). Time-dependent ROC curves exhibited the predictive accuracy of FBG at 3 years (AUC 0.63, 95% CI = 0.55–0.72), 5 years (AUC 0.63, 95% CI = 0.68–0.69), and 7 years (AUC 0.60, 95% CI = 0.57–0.63) (Figure 4C). As shown in Figure 4D, time-dependent ROC curves revealed the predictive accuracy of HDL-C at 3 years (AUC 0.60, 95% CI: 0.52–0.67), 5 years (AUC 0.60, 95% CI: 0.56–0.64), and 7 years (AUC 0.59, 95% CI: 0.56–0.62). The CHG index demonstrated significantly higher AUCs for predicting the risk of CMM compared to TC, FBG, and HDL-C. In multi-community cohort, the predictive accuracy for each variable was as follows: CHG (AUC 0.61, 95% CI = 0.57–0.64), TC (AUC 0.62, 95% CI = 0.58–0.65), FBG (AUC 0.70, 95% CI = 0.67–0.74), HDL-C (AUC 0.59, 95% CI = 0.56–0.63) (Supplementary Figure S2).

### 3.5 Subgroup analysis

**Figure 5:**
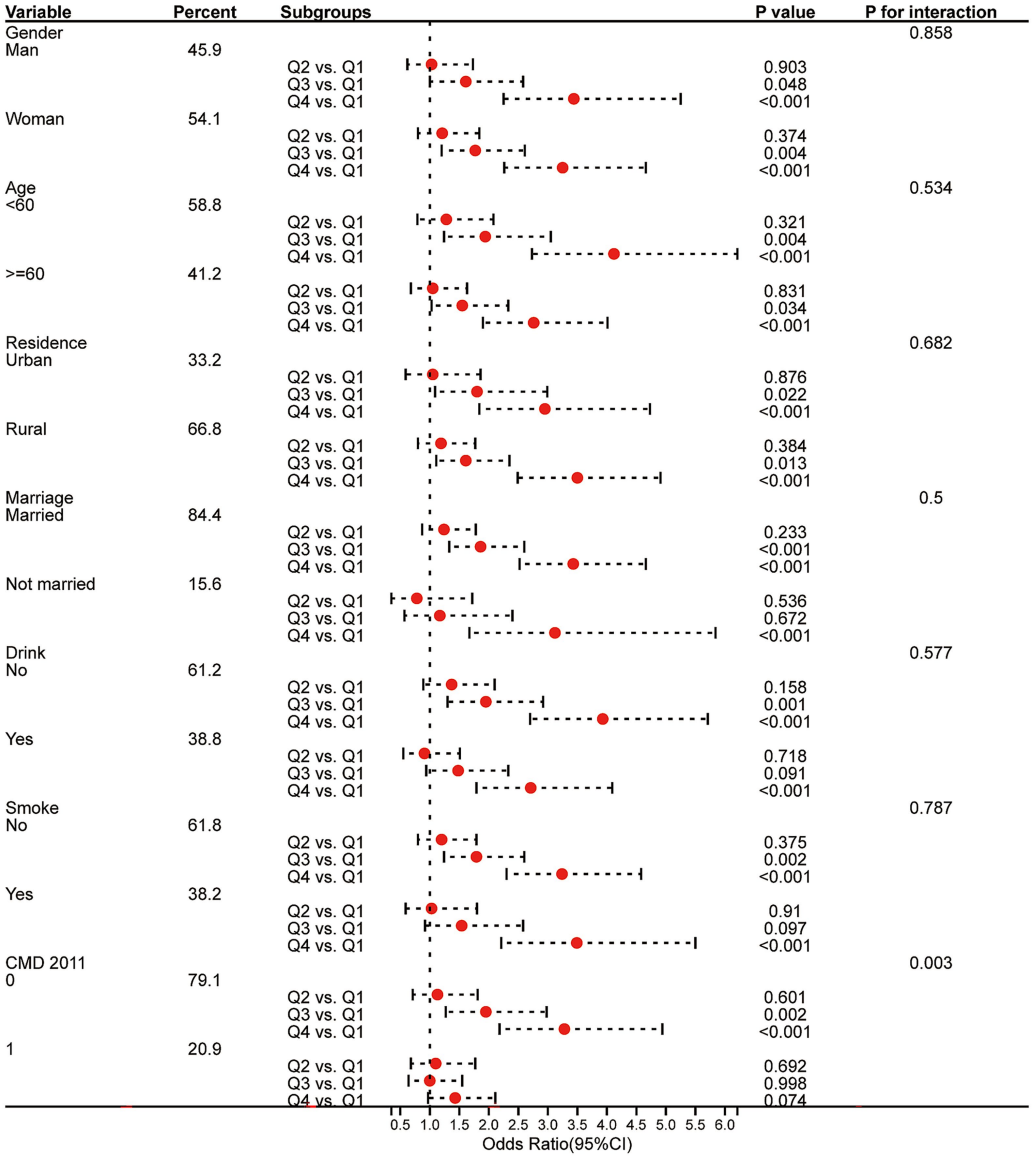
Subgroup analysis of the relationship between CHG and the risk of CMM using logistic proportional hazards regression model *(CHARLS cohort)*. CMD, cardiometabolic disease; OR, odds ratios; CI, confidence interval.

**Figure 6:**
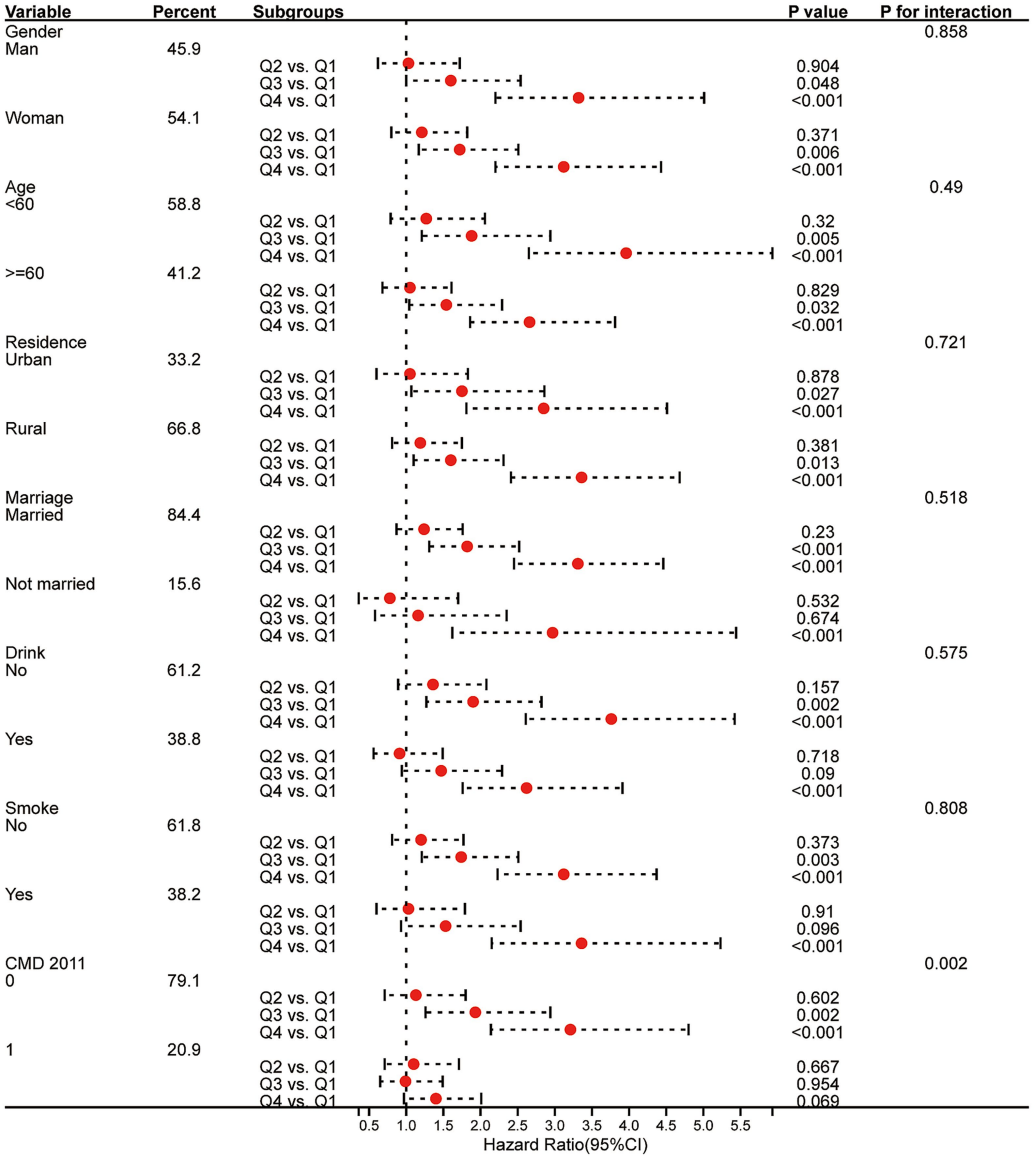
Subgroup analysis of the relationship between CHG and the risk of CMM using Cox proportional hazards regression model *(CHARLS cohort)*. CMD, cardiometabolic disease; HR, hazard ratios; CI, confidence interval.

Furthermore, we conducted subgroup analyses to investigate whether the association between the CHG index and the risk of CMM differs across different subgroups. Logistic regression and Cox regression indicated that compared to Q1, individuals in quartile Q4 had a higher risk of developing CMM in all subgroups, except for individuals with one CMD in 2011 (Figures 5, 6). Both logistic and Cox regression models showed that the difference in the risk of CMM was significant between Q3 and Q1 in all subgroups of gender, age, and residence, and among those who were married, never drank or smoked, and had 0 CMM in 2011 (*p* < 0.05). Furthermore, a significant interactive effect was observed between CMD in 2011 and CHG on the risk of CMM in both Cox proportional hazards regression analysis (*P* for interaction < 0.05) and logistic regression analysis (*P* for interaction < 0.05). Similar results were observed in the multi-community cohort (Supplementary Figure S3).

### 3.6 Sensitivity analyses

Sensitivity analyses were conducted to validate the robustness of the main findings. Subgroup analyses revealed differential associations between the CHG index and CMM according to baseline (2011) CMD status. Therefore, participants of CHARLS cohort were stratified into baseline CMD-free and baseline single-CMD cohorts. Logistic and Cox proportional hazards regression were used to assess the relationships between the CHG index and CMM in each cohort.

In the baseline CMD-free cohort, elevated CHG was significantly associated with in-creased risk of CMM across all models. The risk of CMM showed a significant increase in the Q3 and Q4 groups (OR = 3.91, 95% CI: 2.65–5.77, *p* < 0.001 in model 1; OR = 3.98, 95% CI: 2.68–5.88, *p* < 0.001 in model 2; OR = 3.09, 95% CI: 2.06–4.65, *p* < 0.001 in model 3; Q3 vs. Q1: OR = 1.64, *p* < 0.05, Q4 vs. Q1: OR = 2.56, *p* < 0.001 in model 3) (Supplementary Table S3). Correspondingly, Cox regression showed that higher CHG was significantly associated with an increased risk of CMM. Consistently, the Q3 and Q4 groups showed a significantly in-creased risk of CMM (HR = 2.95, 95% CI = 1.99–4.35, *p* < 0.001; Q3 vs. Q1: HR = 1.62, *p* < 0.05, Q4 vs. Q1: OR = 2.48, *p* < 0.001 in model 3) (Supplementary Table S4).

Conversely, in the single-CMD cohort, only Model 3 showed a significant association (OR = 1.48, 95% CI: 1.07–2.04, *p* < 0.05), and only the increase in the Q4 group showed a significant difference (Q4 vs. Q1: OR = 2.15, *p* < 0.001 in model 3) (Supplementary Table S5). Cox regression showed that higher CHG was significantly associated with an increased risk of CMM, and the Q4 group showed a significant increase (HR = 1.43, 95% CI = 1.08–1.90, *p* < 0.05; Q4 vs. Q1: OR = 1.97, *p* < 0.001 in model 3) (Supplementary Table S6). Although the association be-tween CHG and CMM was stronger in individuals who were CMD-free at baseline, it remained statistically significant in the baseline single-CMD cohort.

## 4 Discussion

This study, based on the CHARLS and multi-community cohorts, explored the associations between the CHG index and CMM both longitudinally and cross-sectionally. The main findings were as follows: (1) Elevated CHG levels demonstrated a significant positive association with the risk of CMM; (2) The association between the CHG index and CMM exhibited nonlinear characteristics and persisted after adjustment for confounding covariates; (3) The CHG index outperformed individual biomarkers (TC, FBG, and HDL-C) in predicting incident CMM, with robust diagnostic performance maintained at 3-year, 5-year, and 7-year follow-up intervals.

In both cohorts, the mean CHG index was consistent with previous reports, supporting the reliability of our findings. Additionally, CHG levels were significantly higher in the CMM group compared to the non-CMM group (Table 1; Supplementary Table S1). As CHG levels increased, the proportion of individuals with CMM also increased (Table 2; Supplementary Table S2). Proposed by Mansoori et al., the CHG index exhibited lower sensitivity but higher specificity for the diagnosis of T2DM. Significantly elevated levels of CHG were observed in the T2DM cohort and the cardiovascular disease (CVD) cohort (*p* < 0.001), indicating its association with CMDs (14, 15). In contrast, we found that the CHG index exhibited lower sensitivity for CMM compared to T2DM (but higher than CVD). In addition, it maintained robust diagnostic efficacy for detecting CMM at different time points.

In CHARLS cohort, during the 7-year follow-up, 491 participants (5.8%) developed incident CMM, consistent with that reported in previous studies (20). In the multi-community cohort, the incidence of CMM was 18.17%, which may be related to the older average age and higher prevalence of basal diseases among participants. Although there were some demographic differences between the two cohorts, both found a positive association between the CHG index and CMM risk (Table 3). This positive association aligns with trends reported for other composite metabolic indices (20, 21). Surrogate IR indexes are widely employed in predicting cardiovascular and metabolic diseases, including CMM (21). In previous comparative analyses, among six surrogate IR indexes, the Chinese visceral adiposity index (CVAI) exhibited optimal diagnostic efficacy (AUC = 0.691) in predicting CMM (20). Another study demonstrated the superiority of the TyG index in predicting mortality in the CMM cohort (AUC = 0.726) (9). The CHARLS cohort results indicated that the CHG index has diagnostic power comparable to CVAI (AUC = 0.67), and outperforms TC, FBG, and HDL-C (Figure 4). However, in the multi-community cohort, FBG (AUC = 0.70) was superior to CHG (AUC = 0.61), which may be attributed to cohort-specific characteristics or may suggest that the CHG index has stronger long-term predictive ability than short-term predictive capacity (Supplementary Figure S2). Additionally, unlike most surrogate IR indexes, CHG was nonlinearly associated with CMM after adjustment for confounding covariates (*P* for nonlinear = 0.002) (Figure 3). Moreover, participants in CHG Q3 exhibited 47% higher risk of CMM (HR: 1.47; 95% CI: 1.06, 1.92; *P* for trend <0.05), while participants in CHG Q4 had 140% increased risk of CMM (HR:2.40; 95% CI: 1.68, 2.94; *P* for trend <0.001) compared to Q1 (Table 4). These risk magnitudes are similar to those conferred by remnant cholesterol and triglycerides (8). Another report from the CHARLS study combining abdominal obesity and non-traditional lipid parameters reported the highest CMM risk among individuals with both conditions (22). Notably, the CHG index had predictive capacity comparable to that of the combination indexes.

The CHG index, calculated from TC, FBG, and HDL-C, provides a composite measure of blood lipid and glucose levels. FBG reflects pancreatic beta-cell function and systemic glucose metabolism, serving as a primary tool for T2DM screening, strongly associated with the risk of complications (23). Elevated blood level of glucose is closely associated with IR. IR, characterized by reduced insulin sensitivity, typically precedes the development of diabetes by years and adversely affects the progression and mortality of CVD/stroke (24, 25). IR induces chronic hyperglycemia, triggering oxidative stress and inflammatory responses. It concurrently disrupts systemic lipid metabolism, leading to dyslipidemia and elevated plasma levels of triglycerides, reduced levels of HDL-C, and increased levels of small dense LDL (26). Furthermore, hyperinsulinemia induced by IR can accelerate the production of fatty acids, inhibit the normal function of insulin, and trigger early atherosclerosis, dyslipidemia, hyperglycemia, and hypertension (27). TC, representing total blood cholesterol across lipoproteins, shows the most significant increase in Chinese populations (28). HDL-C comprises heterogeneous particles (size/composition) with nearly equal amounts of lipids (cholesterol, phospholipids) and proteins, primarily secreted by the liver and intestine (29). Epidemiological data revealed that TC reduction significantly lowers coronary heart disease mortality (30). Basic research revealed that HDL-C protects against cardiovascular diseases by serving as both the acceptor of cholesterol from cells and as the cholesterol carrier in the reverse cholesterol transport pathway (31). Dyslipidemia leads to endothelial injury, oxidative stress, inflammation, and vascular remodeling (32), and has been recognized as a significant risk factor for CMM (33). Increased CHG index signifies the systemic co-occurrence of dyslipidemia and hyperglycemia, both of which are key drivers of CMM. Consistently, an exposome-wide Mendelian randomization study revealed that TC levels and HDL-C levels are the most influential factors genetically associated with a lower risk of CMM, while the study reported that FBG is linked to T2DM and CAD (34). Recent evidence supports that triglyceride-rich lipoproteins and altered glucose metabolism synergistically promote arterial stiffness and metabolic stress (35). Moreover, LDL-lowering through inclisiran has been shown to reduce pulse wave velocity, highlighting the reversibility of metabolic–vascular coupling (36). The CHG index, by integrating glucose and lipid components, likely reflects insulin resistance–driven vascular remodeling, endothelial dysfunction, and subclinical inflammation. Thus, Individuals with high CHG index frequently suffer from IR, inflammation, vascular dysfunction, and CMM.

In this study, the demographic characteristics of the CMM cohort aligned with those of prior reports, showing consistent trends in clinical parameters and medical history. Female gender, advanced age, and urban residency were more common in the CMM population. Although previous studies reported the effects of alcohol consumption and education level on CMM development, these factors exhibited no significant associations (20, 22, 37). Collectively, CMM was associated with older age, female gender, urban residence, lower smoking rates, adverse metabolic/cardiovascular risk markers, and presence of underlying diseases. Notably, participants with elevated CHG index were more likely to have urban residence, not drink, possess abnormal clinical biomarkers, and have histories of diabetes, hypertension, and dyslipidemia. However, there were no significant differences between participants with elevated CHG index and those without elevated CHG index in terms of the history of heart disease and stroke.

Subgroup analysis revealed that the relationship between CHG and CMM varied among different groups in CMD 2011 (Figures 5, 6). Therefore, a sensitivity analysis was conducted, which showed a significant association between CHG and CMM (Supplementary Tables S3–S6), regardless of the presence of CMD at baseline (in 2011). However, compared to participants with a single CMD (OR = 1.48, HR = 1.43, *p* < 0.05) and all participants (OR = 1.97, HR = 1.82, *p* < 0.001), participants who were CMD-free and had high CHG index at baseline were more likely to experience an increase in the risk of CMM (OR = 3.09, HR = 2.95, *p* < 0.001). These results indicate a substantially greater increase in the risk of CMM in CMD-free subjects with high CHG index at baseline.

The present study had several strengths. First, it integrates data from a large, nationally representative prospective cohort and a regionally representative cross-sectional cohort. The prospective cohort provides longitudinal data on incident CMM from 2011 to 2018, while the cross-sectional cohort offers more recent data from multiple communities in 2021, allowing for a comprehensive examination of the relationship between the CHG index and CMM from different perspectives. Second, the 7 years of follow-up enabled the longitudinal assessment of the associations between CHG and CMM, with time-dependent AUC evaluating the predictive capacity of CHG at different time points. Third, subgroup and sensitivity analyses confirmed consistent correlations of CHG and CMM across populations. To our knowledge, this is the first study establishing a positive association between the CHG index and CMM risk, providing robust evidence for the clinical application of the CHG index in predicting CMM.

However, there are several limitations to the current study. First, both cohorts consisted of Chinese older populations, potentially limiting the generalizability of the results. Second, follow-up was conducted at fixed intervals. Although we tracked the exact year of onset for each patient in the CHARLS database, it was impossible to rule out patients who had already developed CMM but were diagnosed later during the follow-up. This could affect the confirmed time of CMM. Third, CMM was diagnosed based on a self-reported physician assessment, which could introduce potential information bias. Additionally, missing data for some participants could introduce selection bias. This should be considered when interpreting and extrapolating results. Despite these limitations, this study extended our previous knowledge of the association between the CHG index and CMM. Future validation of our findings in different ethnic and national populations is necessary.

## 5 Conclusion

In summary, this study demonstrated that a higher CHG level significantly increased the risk of CMM in middle-aged and older Chinese adults. The association was particularly stronger in individuals without underlying CMD. Furthermore, CHG showed consistent and superior diagnostic efficacy for CMM at 3, 5, and 7 years compared to TC, FBG, and HDL-C. These results indicate that CHG serves as a reliable and novel predictor for CMM. Early monitoring and modification of CHG levels may therefore contribute to reducing CMM incidence, improving quality of life, and lowering medical risks in this population.

## Data Availability

The raw data supporting the conclusions of this article will be made available by the authors, without undue reservation.
