# Supplementary material for: Association between the CHG index and cardiometabolic multimorbidity: a nationwide prospective cohort and multi-community cross-sectional study
Source: Front Nutr. 2026 Jan 27;12:1725495. doi: 10.3389/fnut.2025.1725495 (PMC12886036; doi:10.3389/fnut.2025.1725495)
Supplement: Supplementary file 1 [file Supplementary_file_1.docx]

Supplementary Material

**The detail of blood sample testing**

**Blood sample collection, processing, transportation, and storage**

For the blood collection in the national survey we used staff of the Chinese Center for Disease Control and Prevention (China CDC), while for the main survey, including the collection of non-blood biomarkers, we used CHARLS enumerators. We have worked in tandem with China CDC staff in developing the main questionnaire, field protocols, and other aspects of the survey. Working with China CDC had several advantages in this very large country, especially as CHARLS became fully national in scope. Most importantly for this project, working with the China CDC allowed us to collect venous blood instead of relying on dried blood spots. China CDC has a nationwide network and trained staff in all counties. Each county CDC has basic laboratory facilities, even in the most remote regions. Moreover they have experience in large-scale surveys of collecting blood, process- ing samples de-centrally and shipping them to Beijing for analyses at a central lab. The collection of venous blood, blood processing, initial local analysis of complete blood cell counts, and specimen shipment to Beijing for the first national wave of CHARLS was supported by the Chinese National Natural Science Foundation funds under a project entitled, “Health Needs of the Mature and Older Adults in China”. The central laboratory assays of the samples were supported by a competing revision R01 from the National Institute on Aging.

Three tubes of venous blood were collected from each respondent by medically-trained staff from the China CDC, based on a standard protocol. We asked respondents to have fasted overnight, but we took blood even if they were not fasting and we noted their status, which is a variable in the data. Over 92% of respondents who gave blood reported that they were fasting. For most respondents the blood collection was done at centralized locations. In urban areas the district CDC was the central location. In rural areas, the county CDC stations or the town/village health centers were used, whichever was closer. If the location was remote, the village health center was used as the collection center. If none of these were possible, the exam and blood collection was done at the respondent’s home, using the same standards as at the central places (these procedures are covered by a standard CDC manual, “Blood Collection and Handling”).

Identification of the appropriate person was done by matching the name, sex, date of birth, barcode (given by CHARLS enumerators at the main interview) and national identity card number, by county CDC staff. Respondents were told to bring their barcodes and identity cards to the site of the blood collection. The county CDC staff in the field checked the barcodes. The staff had the respondent’s identity card numbers but minus the last four digits. They then used the actual card to fill in the last 4 digits so that we could later verify that the person was the correct one.

The first tube of blood, a 2 mL tube, was used for a complete blood count (CBC) test, including white blood cells, hemoglobin, hematocrit, platelet counts, and mean corpuscular volume. These were measured on automated analyzers available at county CDC stations or town/village health centers. Over 97% of these laboratories participate in a regular assay quality control program.

After collection, these fresh venous blood samples were transported, at 4 ◦C temperature, to either local CDC laboratories or township level hospitals near the study sites. For the overwhelming majority of the respondents (75%) who had fasting blood specimens collected at centralized locations, the CBC was measured within 141 minutes of collection. The median time from collection to CBC assay was 97 minutes.

Second, a 4 mL tube of whole blood was collected to obtain plasma (yield a little under 2 mL of plasma) and buffy coat, which contains predominantly white blood cells. The venous blood was processed and divided into these two components within the same timeframe as the CBC measurement. Transport to the local lab (if transport is required) was at 4 ◦C. After the venous blood was separated into plasma and buffy coat, the plasma was then stored in three 0.5 mL cryovials and the buffy coat in a separate cryovial. These cryovials were then immediately stored frozen at −20 ◦C and transported to the Chinese CDC in Beijing within 2 weeks where they were placed in a deep freezer and stored at −80 ◦C until assay at CMU laboratory.

Finally, one 2mL tube was collected for the HbA1c assay. This 2mL tube of whole blood was stored immediately and during shipment at 4◦C as described above, and transported to the China CDC in Beijing within 2 weeks, where it was placed at −80 ◦C in a deep freezer for the HbA1c assay.

**Handling of blood samples and development of data base**

Two China CDC staff worked full time on maintaining storage of the blood samples; they logged in blood tubes and cryovials as they were received. All tubes and cryovials have barcodes attached to them. For a given respondent each barcode has the same number. When the blood samples were first collected in the field, the barcode was scanned into the CAPI file for the respondent, so that we had a clear link between the barcode number and the CHARLS ID. When the county CDC labs conducted the CBC and when the CMU laboratory conducted its assays, the barcodes were scanned again at the labs in order to make sure we had the correct identification. Files were maintained so that the whereabouts of all samples could be determined at any time. As samples were removed from freezers and sent for assay, files were sent with barcodes and not survey ID numbers. China CDC staff personally carried the samples to the CMU laboratory. Each assay result was merged with the data file by two China CDC staff to ensure accuracy.

**Methods for blood-based bioassays**

In addition to the CBC, which was performed in the laboratories at the county level, we measured high-sensitivity CRP, glycosylated hemoglobin (HbA1c), a lipid panel (total, HDL, LDL cholesterol, and triglycerides), glucose, BUN, creatinine, uric acid, and cystatin C from frozen plasma or whole blood samples. These assays were performed at the Youanmen Center for Clinical Laboratory of Capital Medical University. The assay methods used in this laboratory, coefficients of variation (CVs), and detection limits are summarized below.

This CMU laboratory has regular external quality assessment organized by the Chinese Ministry of Health and conducts assay quality control samples on a daily basis. The Center has been accredited by the Beijing Health Bureau and offers approximately 200 different clinical assays in immunology, chemistry, hematology, virology, and molecular biology. This laboratory has excellent performance during annual evaluation by External Quality Assurance (EQA) Program organized by the National Center for Clinical Laboratories, China Ministry of Health.

The laboratory used quality control (QC) samples daily during the testing of the CHARLS study samples (from February of 2013 to June of 2013). The total numbers of QC samples tested were 117 for blood chemistry tests and lipid panels, 59 for HbA1c assay, and 42 for hsCRP assay. All test results from these QC samples were within the target range (within two standard deviations of mean QC control concentrations).

Perry Hu and Tao Ge checked initial results on a weekly basis to make sure that assays appeared to be within range, that values seem appropriate, and that the quality-control (QC) samples indicate reliability in the process.
