# Supplementary material for: Association between the CHG index and cardiometabolic multimorbidity: a nationwide prospective cohort and multi-community cross-sectional study
Source: Front Nutr. 2026 Jan 27;12:1725495. doi: 10.3389/fnut.2025.1725495 (PMC12886036; doi:10.3389/fnut.2025.1725495)
Supplement: Supplementary file 2 [file Supplementary_file_2.docx]

Supplementary Material

**Table S1 Baseline characteristics of the study participants according to cardiometabolic multimorbidity outcomes (****Multi-community cohort)**

| **Characteristic** | **Overall** | **Non-CMM group** | **CMM group** | ***P*-value** |
| --- | --- | --- | --- | --- |
| **N** | 1734 | 1419 | 315 |  |
| **Age, year** | 66.14±10.23 | 65.41±10.39 | 69.42±8.79 | ＜0.001 |
| **Man, n(％)** | 667(38.5) | 535(37.7) | 132(41.9) | 0.17 |
| **Current married, n(％)** | 1514(87.3) | 1255(88.4) | 259(82.2) | ＜0.001 |
| **Educational level, n(％)** |  |  |  | 0.01 |
| **Primary school or lower** | 80(4.6) | 62(4.6) | 18(5.7) |  |
| **Secondary school** | 914(52.7) | 728(51.3) | 186(59.0) |  |
| **High school** | 740(42.7) | 629(44.3) | 111(35.2) |  |
| **Smoking, n(％)** | 295(17.0) | 228(16.1) | 67(21.3) | 0.03 |
| **Drinking, n(％)** | 360(20.8) | 295(20.8) | 65(20.6) | 0.95 |
| **Basal Diabetes, n(％)** | 384(20.1) | 115(8.1) | 233(74.0) | ＜0.001 |
| **Basal Heart disease, n(％)** | 242(14.0) | 157(11.1) | 85(27.0) | ＜0.001 |
| **Basal Stroke, n(％)** | 167(9.6) | 45(3.4) | 119(37.8) | ＜0.001 |
| **Basal Hypertension, n(％)** | 866(49.9) | 557(39.3) | 309(98.1) | ＜0.001 |
| **Basal Dyslipidemia, n(％)** | 822(47.4) | 630(44.4) | 192(61.0) | ＜0.001 |
| **BMI, kg/m^2^** | 24.73±4.25 | 24.58±4.14 | 25.43±4.66 | 0.001 |
| **SBP, mmHg** | 133.79±17.61 | 133.04±17.60 | 137.16±17.29 | ＜0.001 |
| **DBP, mmHg** | 79.89±10.24 | 79.87±10.17 | 79.97±10.55 | 0.87 |
| **FBG, mg/dL** | 97.20(88.20,115.20) | 95.40(88.20,109.80) | 115.20(97.20,140.40) | ＜0.001 |
| **TC, mg/dL** | 168.73(141.64,195.53) | 151.32(120.94,180.34) | 171.44(145.13,197.76) | ＜0.001 |
| **TG, mg/dL** | 116.95(85.06,165.02) | 115.18(84.17,164.80) | 123.15(90.37,169.23) | 0.07 |
| **LDL-C, mg/dL** | 91.72(69.66,109.91) | 80.11(59.60,99.07) | 91.72(72.37,111.46) | ＜0.001 |
| **HDL-C, mg/dL** | 52.63(44.51,62.31) | 53.41(44.89,63.08) | 48.76(42.18,58.05) | ＜0.001 |
| **HbA1c, ％** | 5.50(5.20,5.90) | 5.40(5.20,5.80) | 6.10(5.50,6.90) | ＜0.001 |
| **CHG** | 5.06(4.84,5.31) | 5.03(4.82,5.28) | 5.18(4.91,5.46) | ＜0.001 |

Date are presented as median (25th to 75th interquartile range) or n (%)

CHG: Cholesterol, High density lipoprotein, and Glucose index; CMM: cardiometabolic multimorbidity; BMI: body mass index; SBP: systolic blood pressure; DBP: diastolic blood pressure; hsCRP: high-sensitivity C-reactive protein; FBG: fasting plasma glucose; TC: total cholesterol; TG: triglycerides; LDL-C: low-density lipoprotein cholesterol; HDL-C: high-density lipoprotein cholesterol; HbA1c: glycosylated hemoglobin A1c; UA: uric acid; BUN: blood urea nitrogen

**Table S2 Baseline characteristics of participants according to the quartiles of CHG(Multi-community cohort)**

| **Characteristic** | **CHG quartiles** |  |  |  |  | ***P*-value** |
| --- | --- | --- | --- | --- | --- | --- |
|  | **Overall** | **Q1** | **Q2** | **Q3** | **Q4** |  |
| **N** | 1734 | 433 | 434 | 434 | 433 |  |
| **Age, year** | 66.14±10.23 | 66.66±10.68 | 65.61±10.45 | 66.11±9.75 | 66.17±10.03 | 0.52 |
| **Man, n(％)** | 667(38.5) | 157(36.3) | 161(37.1) | 174(40.1) | 175(40.4) | 0.49 |
| **Current married, n(％)** | 1514(87.3) | 371(85.7) | 377(86.9) | 385(88.7) | 381(88.0) | 0.56 |
| **Educational level, n(％)** |  |  |  |  |  | 0.70 |
| **Primary school or lower** | 80(4.6) | 15(3.5) | 19(4.4) | 25(5.8) | 21(4.8) |  |
| **Secondary school** | 914(52.7) | 223(51.5) | 228(52.5) | 229(52.8) | 234(54.0) |  |
| **High school** | 740(42.7) | 195(45.0) | 187(43.1) | 180(41.5) | 178(41.1) |  |
| **Smoking, n(％)** | 295(17.0) | 69(15.9) | 69(15.9) | 77(17.7) | 80(18.5) | 0.67 |
| **Drinking, n(％)** | 360(20.8) | 80(18.5) | 82(18.9) | 102(23.5) | 96(22.2) | 0.19 |
| **Basal Diabetes, n(％)** | 348(20.1) | 48(11.1) | 63(14.5) | 74(17.1) | 163(37.6) | ＜0.001 |
| **Basal Heart disease, n(％)** | 242(14.0) | 69(15.9) | 63(14.5) | 60(13.8) | 50(11.5) | 0.31 |
| **Basal Stroke, n(％)** | 167(9.6) | 47(10.9) | 52(12.0) | 32(7.4) | 36(8.3) | 0.08 |
| **Basal Hypertension, n(％)** | 866(49.9) | 200(46.2) | 202(46.5) | 228(52.5) | 236(54.5) | 0.03 |
| **Basal Dyslipidemia, n(％)** | 822(47.4) | 192(44.3) | 212(48.8) | 199(45.9) | 219(50.6) | 0.25 |
| **BMI, kg/m^2^** | 24.73±4.25 | 23.77±4.07 | 24.50±3.82 | 25.12±4.64 | 25.55±4.23 | ＜0.001 |
| **SBP, mmHg** | 133.79±17.61 | 132.64±17.20 | 133.29±17.94 | 134.41±16.63 | 134.80±18.59 | 0.26 |
| **DBP, mmHg** | 79.89±10.24 | 79.47±10.20 | 79.09±10.08 | 79.93±10.71 | 81.06±9.88 | 0.03 |
| **FBG, mg/dL** | 107.01±45.88 | 86.32±14.72 | 96.90±13.76 | 106.28±13.58 | 138.57±78.25 | ＜0.001 |
| **TC, mg/dL** | 170.97±45.49 | 148.37±37.24 | 167.45±45.09 | 176.52±43.97 | 191.60±44.23 | ＜0.001 |
| **TG, mg/dL** | 138.00±15.59 | 99.49±14.98 | 122.24±16.63 | 147.16±13.67 | 183.10±12.83 | ＜0.001 |
| **LDL-C, mg/dL** | 91.69±32.02 | 74.61±25.97 | 86.14±26.14 | 96.23±30.03 | 109.77±34.35 | ＜0.001 |
| **HDL-C, mg/dL** | 54.75±15.05 | 62.53±16.16 | 57.07±14.61 | 51.89±13.41 | 47.48±11.23 | ＜0.001 |
| **HbA1c, ％** | 5.72±1.00 | 5.41±0.63 | 5.53±0.65 | 5.65±0.76 | 6.29±1.46 | ＜0.001 |
| **CMM, n(％)** | 315(18.2) | 56(12.9) | 70(16.1) | 69(15.9) | 120(27.7) | ＜0.001 |

Date are presented as median (25th to 75th interquartile range) or n (%)

CHG: Cholesterol, High density lipoprotein, and Glucose index; CMM: cardiometabolic multimorbidity; BMI: body mass index; SBP: systolic blood pressure; DBP: diastolic blood pressure; hsCRP: high-sensitivity C-reactive protein; FBG: fasting plasma glucose; TC: total cholesterol; TG: triglycerides; LDL-C: low-density lipoprotein cholesterol; HDL-C: high-density lipoprotein cholesterol; HbA1c: glycosylated hemoglobin A1c; UA: uric acid; BUN: blood urea nitrogen

**Table S3** Logistic regression analysis of baseline CHG and CMM in baseline CMD-free Participants **(CHARLS cohort )**

|  | **OR (95% CI)** | | |
| --- | --- | --- | --- |
|  | **Model 1^a^** | **Model 2^b^** | **Model 3^c^** |
| CHG, Continuous | 3.91(2.65-5.77)*** | 3.98(2.68-5.88)*** | 3.09(2.06-4.65)*** |
| CHG, Categories |  |  |  |
| Q1 | Reference | Reference | Reference |
| Q2 | 1.13(0.71-1.81) | 1.14(0.71-1.82) | 1.03(0.64-1.66) |
| Q3 | 1.95(1.28-3.01)** | 1.97(1.29-3.06)** | 1.64(1.07-2.56)* |
| Q4 | 3.28(2.20-5.00)*** | 3.33(2.22-5.09)*** | 2.56(1.69-3.95)*** |

^a^ Model 1 unadjusted for any covariates

^b^ Model 2 adjusted for gender, age, residence, marital status, education level, smoking status, and drinking status

^c^ Model 3 adjusted for gender, age, residence, marital status, education level, smoking status, drinking status, diabetes, heart disease, stroke, hypertension, dyslipidemia, diabetes medications, heart disease medications, stroke medications, status of CMD 2011, SBP, DBP, and BMI. CHG: Cholesterol, High density lipoprotein, and Glucose index.

*P < 0.05. **P < 0.01. ***P < 0.001.

**Table S4** Cox proportional hazards regression model analysis of baseline CHG and CMD in baseline CMD-free Participants **(CHARLS cohort )**

|  | **HR (95% CI)** | | |
| --- | --- | --- | --- |
|  | **Model 1^a^** | **Model 2^b^** | **Model 3^c^** |
| CHG, Continuous | 3.78(2.60-5.51)*** | 3.82(2.62-5.59)*** | 2.95(1.99-4.35)*** |
| CHG, Categories |  |  |  |
| Q1 | Reference | Reference | Reference |
| Q2 | 1.13(0.71-1.80) | 1.13(0.71-1.80) | 1.03(0.65-1.64) |
| Q3 | 1.93(1.27-2.94)** | 1.95(1.28-2.98)** | 1.62(1.06-2.48)* |
| Q4 | 3.21(2.14-4.80)*** | 3.24(2.16-4.86)*** | 2.48(1.64-3.75)*** |

^a^ Model 1 unadjusted for any covariates

^b^ Model 2 adjusted for gender, age, residence, marital status, education level, smoking status, and drinking status

^c^ Model 3 adjusted for gender, age, residence, marital status, education level, smoking status, drinking status, diabetes, heart disease, stroke, hypertension, dyslipidemia, diabetes medications, heart disease medications, stroke medications, status of CMD 2011, SBP, DBP, and BMI. CHG: Cholesterol, High density lipoprotein, and Glucose index.

*P < 0.05. **P < 0.01. ***P < 0.001.

**Table S5** Logistic regression analysis of baseline CHG and CMM in baseline single-CMD Participants **(CHARLS cohort )**

|  | **OR (95% CI)** | | |
| --- | --- | --- | --- |
|  | **Model 1^a^** | **Model 2^b^** | **Model 3^c^** |
| CHG, Continuous | 0.99(0.76-1.28) | 0.99(1.76-1.29) | 1.48(1.07-2.04)* |
| CHG, Categories |  |  |  |
| Q1 | Reference | Reference | Reference |
| Q2 | 1.10(0.69-1.78) | 1.07(0.66-1.73) | 1.13(0.69-1.84) |
| Q3 | 1.00(0.65-1.56) | 0.97(0.63-1.53) | 1.22(0.78-1.95) |
| Q4 | 1.43(0.97-2.13) | 1.39(0.94-2.08) | 2.15(1.41-3.35)*** |

^a^ Model 1 unadjusted for any covariates

^b^ Model 2 adjusted for gender, age, residence, marital status, education level, smoking status, and drinking status

^c^ Model 3 adjusted for gender, age, residence, marital status, education level, smoking status, drinking status, diabetes, heart disease, stroke, hypertension, dyslipidemia, diabetes medications, heart disease medications, stroke medications, status of CMD 2011, SBP, DBP, and BMI. CHG: Cholesterol, High density lipoprotein, and Glucose index.

*P < 0.05. **P < 0.01. ***P < 0.001.

**Table S6** Cox proportional hazards regression model analysis of baseline CHG and CMD in baseline single-CMD Participants **(CHARLS cohort )**

|  | **HR (95% CI)** | | |
| --- | --- | --- | --- |
|  | **Model 1^a^** | **Model 2^b^** | **Model 3^c^** |
| CHG, Continuous | 0.99(0.78-1.26) | 1.00(0.78-1.27) | 1.43(1.08-1.90)* |
| CHG, Categories |  |  |  |
| Q1 | Reference | Reference | Reference |
| Q2 | 1.10(0.71-1.71) | 1.08(0.70-1.68) | 1.13(0.73-1.76) |
| Q3 | 0.99(0.65-1.49) | 0.97(0.64-1.46) | 1.18(0.78-1.80) |
| Q4 | 1.40(0.97-2.01) | 1.36(0.95-1.96) | 1.97(1.34-2.89)*** |

^a^ Model 1 unadjusted for any covariates

^b^ Model 2 adjusted for gender, age, residence, marital status, education level, smoking status, and drinking status

^c^ Model 3 adjusted for gender, age, residence, marital status, education level, smoking status, drinking status, diabetes, heart disease, stroke, hypertension, dyslipidemia, diabetes medications, heart disease medications, stroke medications, status of CMD 2011, SBP, DBP, and BMI. CHG: Cholesterol, High density lipoprotein, and Glucose index.

*P < 0.05. **P < 0.01. ***P < 0.001.

**
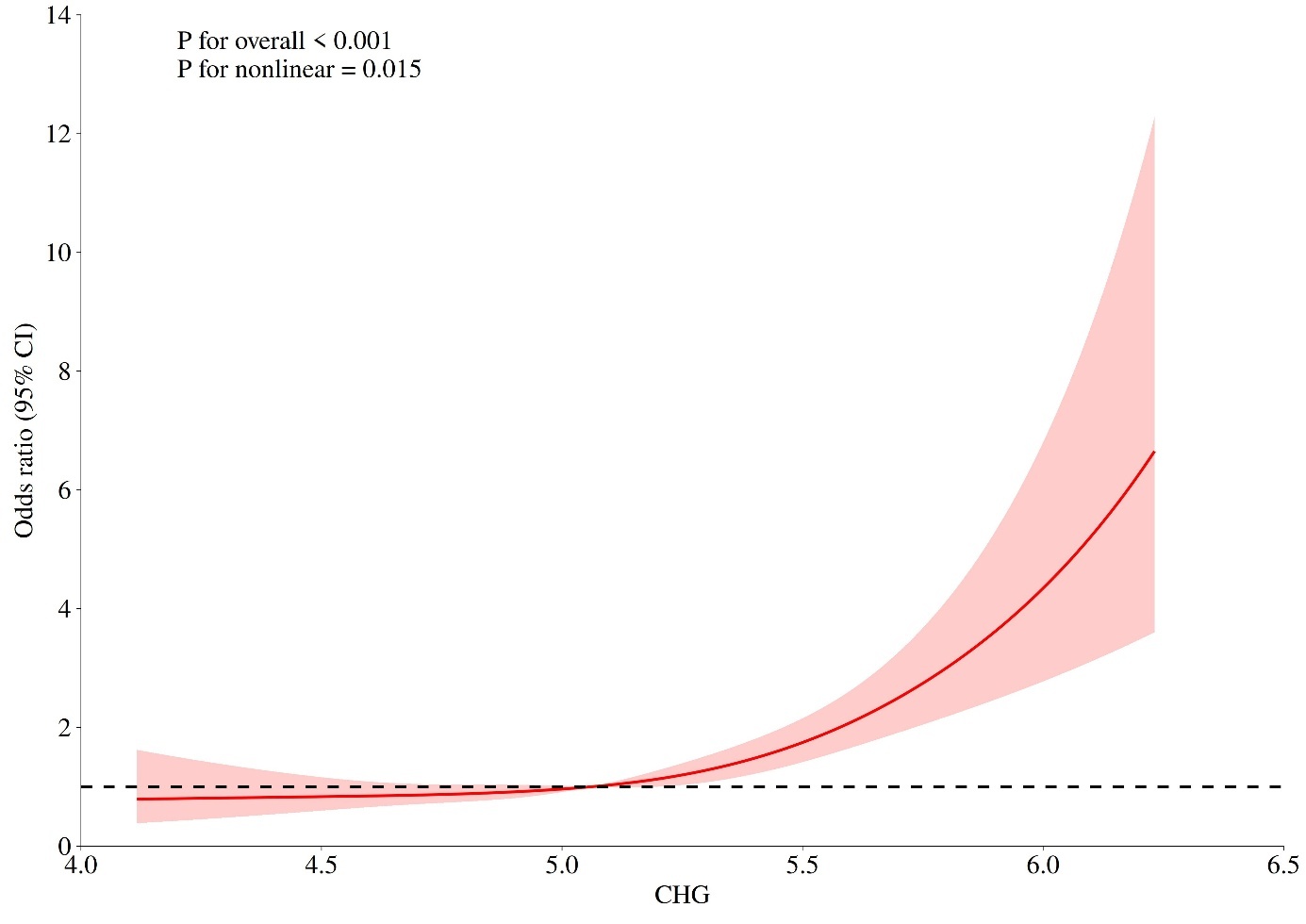
**

**Fig. S1** Restricted cubic spline curves for CMM according to CHG of using logistic regression analysis (**Multi-community cohort**)

adjustment for gender, age, residence, marital status, education level, smoking status, drinking status, SBP, DBP, and BMI.

Abbreviation: CHG, Cholesterol, High density lipoprotein, and Glucose index; CMM, cardiometabolic multimorbidity.

**
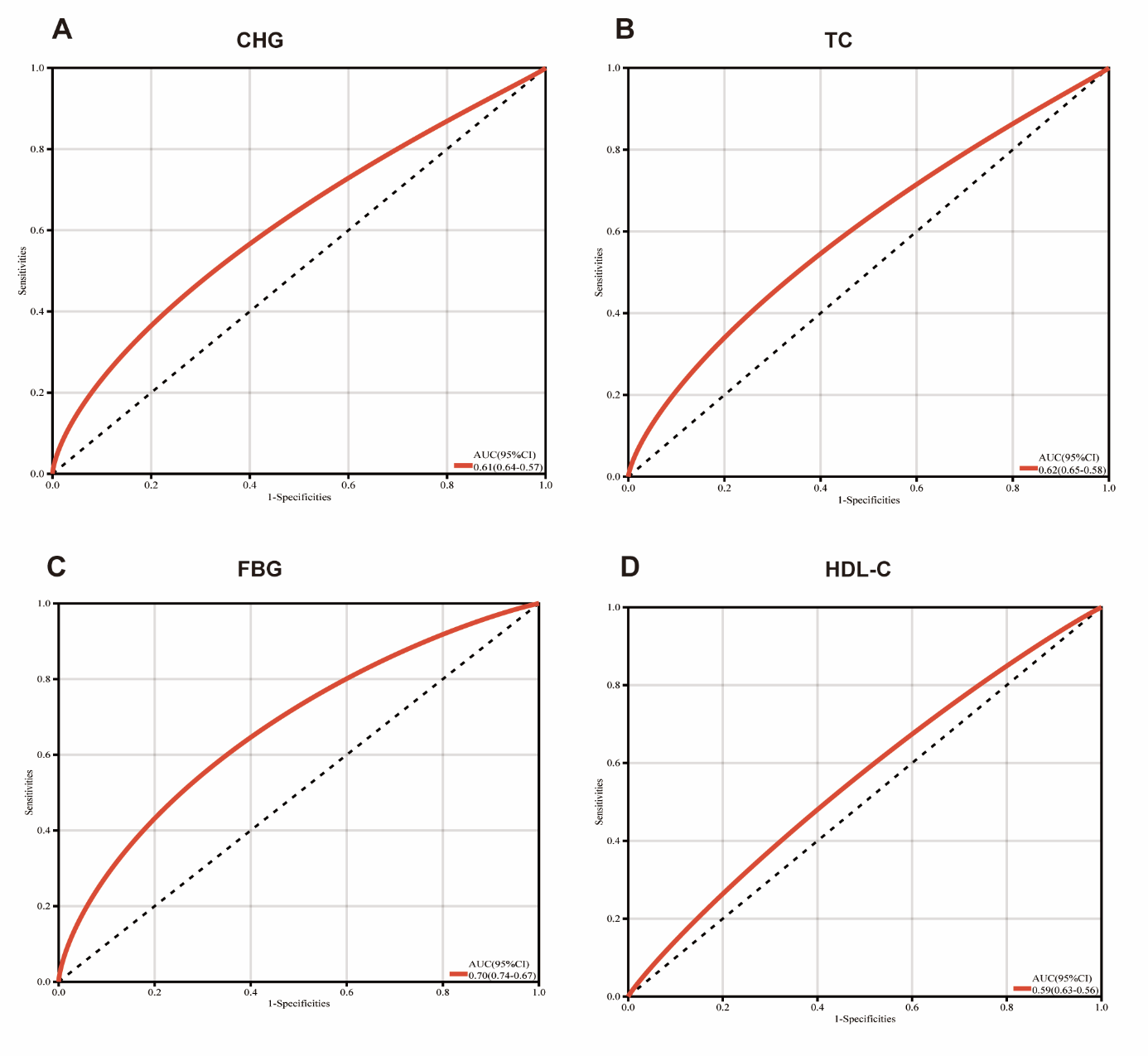
**

**Fig. S2** Receiver-operating characteristic curves for prediction of CMM(**Multi-community cohort**)

Abbreviation: AUC, area under the curve; CHG, Cholesterol, High density lipoprotein, and Glucose index; TC: total cholesterol; FBG: fasting plasma glucose; HDL-C: high-density lipoprotein cholesterol.


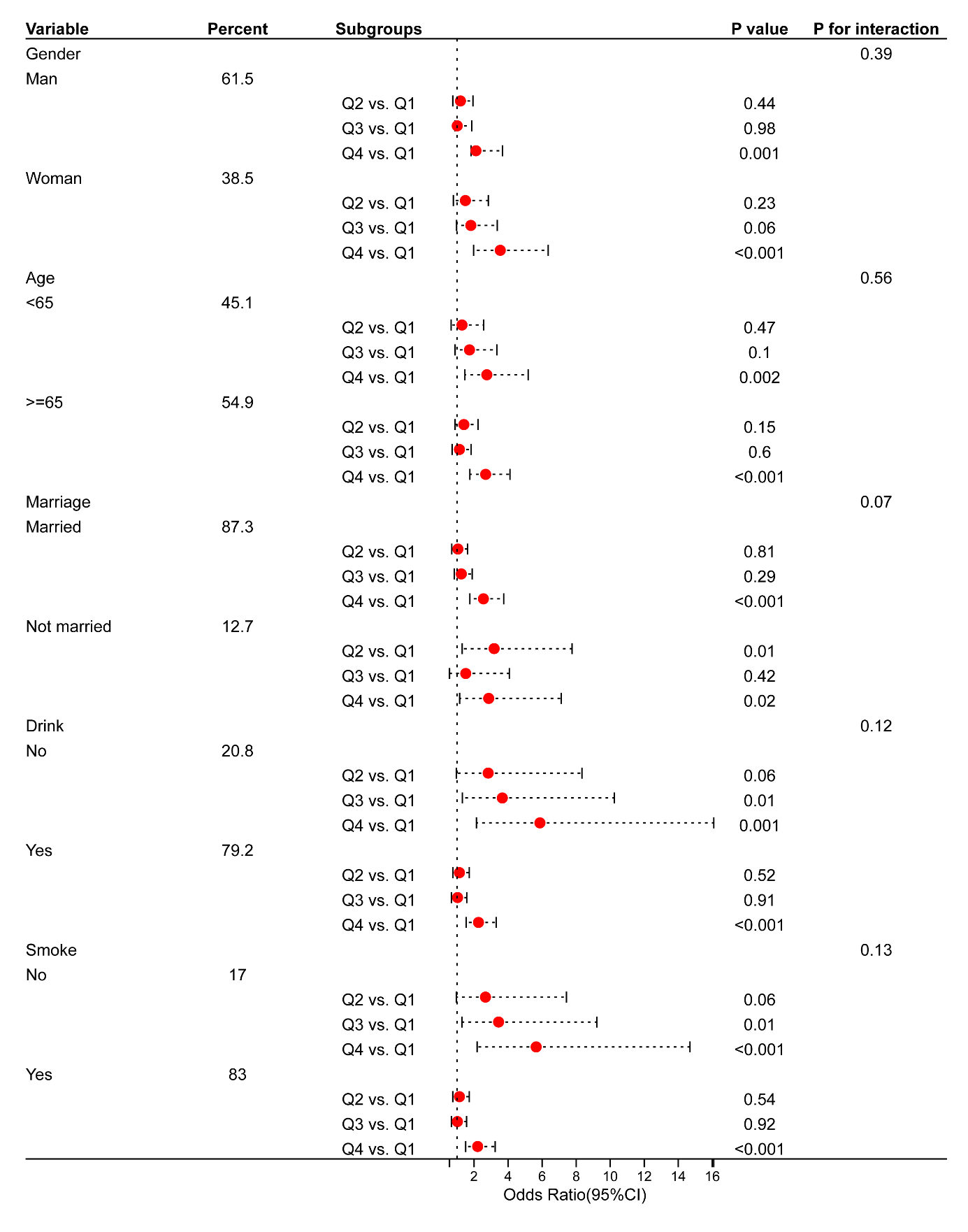


**Fig. S3** Subgroup analysis of the relationship between CHG and the risk of CMM using logistic proportional hazards regression model(**Multi-community cohort**)

Abbreviation: CMM, cardiometabolic multimorbidity; OR, odds ratios; CI, Confidence interval
